# Supplementary material for: Mitogenomic phylogeny of Callithrix with special focus on human transferred taxa
Source: BMC Genomics. 2021 Apr 6;22:239. doi: 10.1186/s12864-021-07533-1 (PMC8025498; doi:10.1186/s12864-021-07533-1)
Supplement: Supplementary file 2 — Additional file 2: Figure S1. Maximum-likelihood (ML) tree showing phylogenetic relationships among Callithrix haplotypes as calculated from mitogenome sequences. Numbers at nodes indicate bootstrap support for a given node, otherwise node bootstrap support was 100%. Haplotype colors at tips correspond to the ‘Species and Hybrid Phenotypes’ legend, and indicate phenotypes associated with each given haplotype. Figure S2. Bayesian tree showing phylogenetic relationships among Callithrix species and hybrid haplotypes from mitogeomes sequences. Numbers at nodes indicate posterior probability for a given node, otherwise node posterior probability was 1. Haplotype colors at tips correspond to the ‘Species and Hybrid Phenotypes’ legend, and indicate phenotypes associated with each given haplotype. Figure S3. BEAST tree showing phylogenetic relationships and divergence ages in million years (Ma) among Callithrix haplotypes and other New World primates as calculated from mitogeome sequences. Major nodes are identified by capital letters, and blue bars at all nodes indicate 95% highest posterior densities (HPD) of divergence times. Haplotype colors at tips correspond to the ‘Species and Hybrid Phenotypes’ legend, and indicate phenotypes associated with each given haplotype. [file 12864_2021_7533_MOESM2_ESM.zip › FigureS2.pdf]

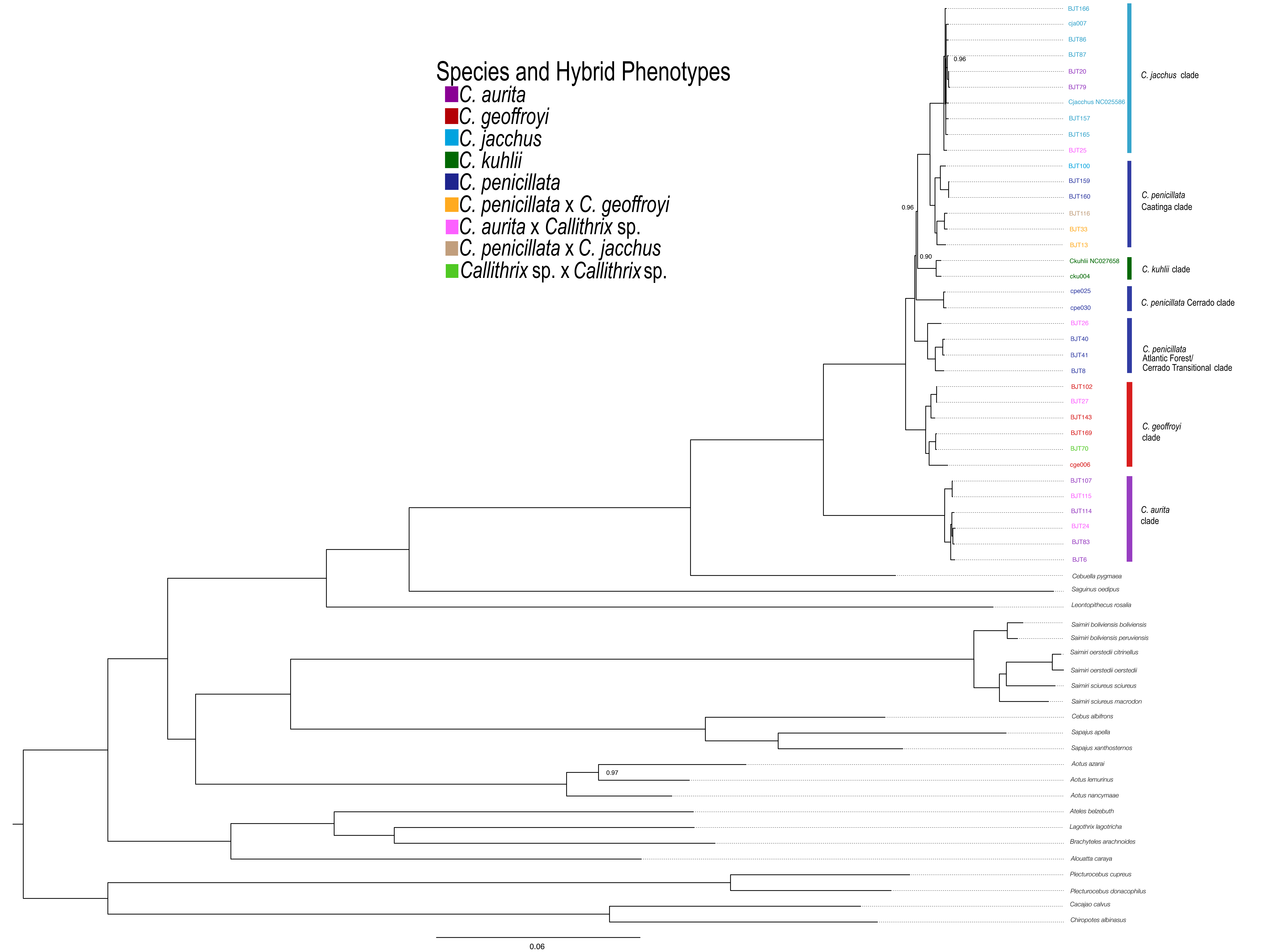

Species and Hybrid Phenotypes
C. aurita
C. geoffroyi
C. jacchus
C. kuhlii
C. penicillata
C. penicillata x C. geoffroyi
C. aurita x Callithrix sp.
C. penicillata x C. jacchus
Callithrix sp. x Callithrix sp.
BJT166
cja007
BJT86
BJT87
0.96
BJT20
BJT79
Cjacchus NC025586
BJT157
BJT165
BJT25
C. jacchus clade
BJT100
BJT159
BJT160
BJT116
BJT33
BJT13
0.96
C. penicillata Caatinga clade
0.90
Ckuhlii NC027658
cku004
C. kuhlii clade
cpe025
cpe030
C. penicillata Cerrado clade
BJT26
BJT40
BJT41
BJT8
C. penicillata Atlantic Forest/ Cerrado Transitional clade
BJT102
BJT27
BJT143
BJT169
BJT70
cge006
C. geoffroyi clade
BJT107
BJT115
BJT114
BJT24
BJT83
BJT6
C. aurita clade
Cebuella pygmaea
Saguinus oedipus
Leontopithecus rosalia
Saimiri boliviensis boliviensis
Saimiri boliviensis peruviensis
Saimiri oerstedii citrinellus
Saimiri oerstedii oerstedii
Saimiri sciureus sciureus
Saimiri sciureus macrodon
Cebus albifrons
Sapajus apella
Sapajus xanthosternos
0.97
Aotus azarai
Aotus lemurinus
Aotus nancymaae
Ateles belzebuth
Lagothrix lagotricha
Brachyteles arachnoides
Alouatta caraya
Plecturocebus cupreus
Plecturocebus donacophilus
Cacajao calvus
Chiropotes albinasus
0.06
