# Supplementary figures and images for: Mitogenomic phylogeny of Callithrix with special focus on human transferred taxa
Source: BMC Genomics. 2021 Apr 6;22:239. doi: 10.1186/s12864-021-07533-1 (PMC8025498; doi:10.1186/s12864-021-07533-1)

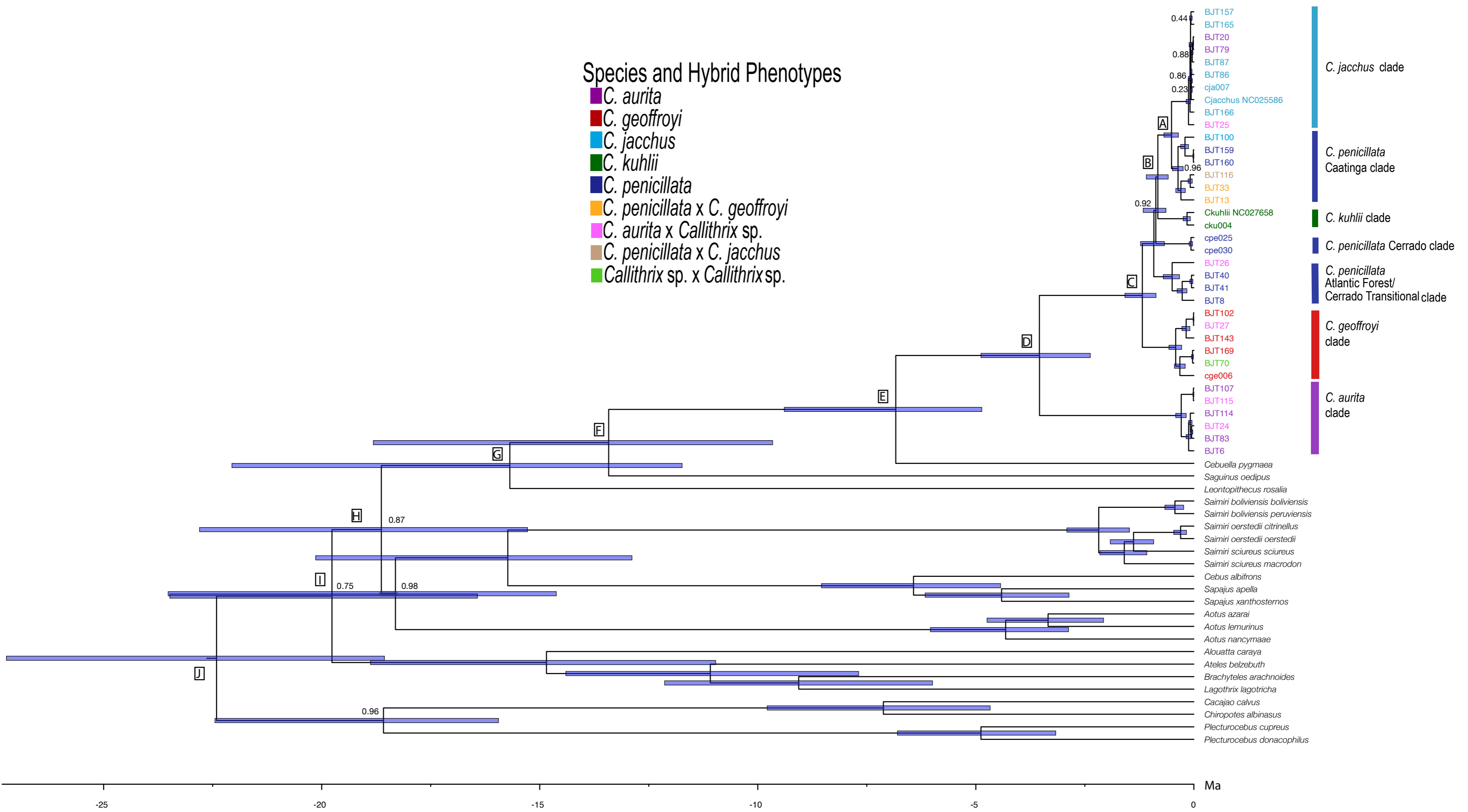

Supplement: Supplementary file 2 — Additional file 2: Figure S1. Maximum-likelihood (ML) tree showing phylogenetic relationships among Callithrix haplotypes as calculated from mitogenome sequences. Numbers at nodes indicate bootstrap support for a given node, otherwise node bootstrap support was 100%. Haplotype colors at tips correspond to the ‘Species and Hybrid Phenotypes’ legend, and indicate phenotypes associated with each given haplotype. Figure S2. Bayesian tree showing phylogenetic relationships among Callithrix species and hybrid haplotypes from mitogeomes sequences. Numbers at nodes indicate posterior probability for a given node, otherwise node posterior probability was 1. Haplotype colors at tips correspond to the ‘Species and Hybrid Phenotypes’ legend, and indicate phenotypes associated with each given haplotype. Figure S3. BEAST tree showing phylogenetic relationships and divergence ages in million years (Ma) among Callithrix haplotypes and other New World primates as calculated from mitogeome sequences. Major nodes are identified by capital letters, and blue bars at all nodes indicate 95% highest posterior densities (HPD) of divergence times. Haplotype colors at tips correspond to the ‘Species and Hybrid Phenotypes’ legend, and indicate phenotypes associated with each given haplotype. [file 12864_2021_7533_MOESM2_ESM.zip › FigureS3.pdf]
